# Allelic Polymorphism Determines Surface Expression or Intracellular Retention of the Human NK Cell Receptor KIR2DL5A (CD158f)

**DOI:** 10.3389/fimmu.2016.00698

**Published:** 2017-01-17

**Authors:** Elisa Cisneros, Ernesto Estefanía, Carlos Vilches

**Affiliations:** ^1^Immunogenetics and Histocompatibility, Instituto de Investigación Sanitaria Puerta de Hierro, Madrid, Spain

**Keywords:** natural killer cells, repertoire development, KIR, NK-cell receptors, allelic polymorphism

## Abstract

KIR2DL5 (CD158f) is the most recently identified inhibitory member of human killer-cell Ig-like receptors (KIRs), which enable NK cells to sense self-HLA. Unlike KIR2DL1–3, recognizing HLA-C allotypes through Ig-like domains of the D1–D2 type, KIR2DL5 shares a D0–D2 configuration with KIR2DL4, and its ligands have not been identified. KIR2DL5 is encoded by two paralogous genes displaying copy number variation and allelic polymorphism—*KIR2DL5A* and *KIR2DL5B*. UP-R1 mAb, raised against the common allele KIR2DL5A*001, enables specific KIR2DL5 detection. However, not every *KIR2DL5*^+^ individual has NK cells staining with UP-R1, discrepancy explained in part by epigenetically silent *KIR2DL5B* alleles with a distinctive substitution in a promoter RUNX-binding site. Furthermore, we show here that the transcribed allele KIR2DL5A*005, second most common of its locus, fails to confer NK cells UP-R1 reactivity, phenotype explained by inefficacious transport of its product to the cell surface. Two amino acid substitutions distinguish the KIR2DL5A*005 and *001 coding regions. Western blot, flow cytometry, and confocal microscopy analyses of cells transfected with tagged constructs demonstrate that a serine substitution for glycine-174, conserved in most KIR, is mainly responsible for KIR2DL5A*005 intracellular retention, and it also affects mAb recognition. In contrast, substitution of aspartate for asparagine 152 has only a minor effect on surface expression, despite destroying an otherwise conserved N-glycosylation site. Our results help to explain the variable expression profile of *KIR2DL5*^+^ subjects and indicate that functional polymorphisms in both its promoter and its coding regions are critical for understanding the KIR2DL5 role in immunity and its importance for human health.

## Introduction

Several inhibitory members of the human killer-cell Ig-like receptors (KIRs) family recognize allotypes of classical HLA class I molecules, and they enable NK cells to monitor, through the missing-self mechanism, selective subversion of antigen presentation in infected and tumor cells (1, 2). Besides, other KIRs have less well-defined functions in immunity (3). Among these is KIR2DL5 (CD158f), which is identified in surveys of the human genome years after the other KIR (4, 5). Like the divergent KIR 2DL4, 2DL5 has Ig-like domains homologous to the D0 and D2 of three-domain KIR and is conserved in several hominoid species. But unlike KIR 2DL4, 2DL5 shares with HLA-C-specific KIR 2DL1–3 (i.e., those with D1–D2 domains) a clearly inhibitory capacity and clonal expression on NK cells and T-lymphocytes (6). The ligand recognized by KIR2DL5 remains, however, elusive.

KIR2DL5 has a complex genetics due to copy number variation and allelic polymorphism (7). It is encoded in the human genome by two closely related genes—*KIR2DL5A* in the telomeric segment of KIR haplotypes and *KIR2DL5B* in the centromeric interval (8, 9). Each of these loci is present or absent in different common haplotypes. Moreover, *KIR2DL5A* has undergone further duplication in some rare haplotypes (10–12). *KIR2DL5A* and *B* are represented by 15 and 28 alleles, respectively, in the Immuno Polymorphism Database release 2.6.0 (5 and 13 alleles, without taking into account non-coding polymorphisms) (13). *KIR2DL5A* is most commonly represented by allele 2DL5A*001, followed in frequency by 2DL5A*005, whereas the centromeric locus is usually represented by allele 2DL5B*002 and also by 2DL5B*006 in Black populations [Ref. (11, 14–17) and our own unpublished data].

Allelic polymorphism affects the coding and the non-coding regions of *KIR2DL5* (7, 13). Of particular functional relevance is one polymorphic G > A substitution at nucleotide 97 before the initiation codon that destroys a RUNX-binding site conserved in the proximal promoter of most *KIR*. Such change correlates exactly with complete epigenetic silencing of the common alleles of *KIR2DL5B* and the *KIR3DP1* pseudogene, while all known *KIR2DL5A* and a few *KIR2DL5B* and *KIR3DP1* alleles have intact RUNX-binding sites and are clonally transcribed (8, 14, 18–20).

Also of interest are two linked dimorphisms in the exon coding for the membrane-proximal D2 Ig-like domain (codons 152 and 174), since they sort all alleles of both the centromeric and the telomeric *KIR2DL5* loci into two mutually exclusive groups (Table 1). Asparagine and glycine are seen in the common 2DL5A*001 allele, in 2DL5B*006, and in other less-common alleles of both loci, besides being conserved in virtually all KIR, while 2DL5A*005 on the telomeric side, the dominant centromeric allele 2DL5B*002 and others encode aspartate and serine at those positions (7). No *KIR2DL5* allele with a mixed motif has been described.

**Table 1:** **Common dimorphisms of the *KIR2DL5* coding and promoter regions**.

		Representative alleles	
Locus	Promoter nucleotide -97	Asn152-Gly174	Asp152-Ser174
*KIR2DL5A*	G	*001	*005
*KIR2DL5B*	A/G^a^	*006	*002

*Guanidine at nucleotide -97 correlates with clonal transcription; adenosine with complete silencing; Asn152-Gly174 and Asp152-Ser174 with cell surface and intracellular expression, respectively*.

^a^Each of KIR2DL5B*006 and *002 comprises multiple alleles coding for identical polypeptides, but differing from each other by changes in nucleotide -97 (besides other non-coding or synonymous substitutions) (13).

Generation of a specific monoclonal antibody, UP-R1, enabled us to substantiate KIR2DL5 protein expression on NK cells and to characterize its biochemical and functional features. However, characterization was limited to NK cells expressing the dominant allele KIR2DL5A*001, against which the mAb was raised (6). Some discrepancies between genotyping and flow cytometry studies were subsequently observed, and they suggested that the aforementioned polymorphisms in the KIR2DL5 primary structure might somehow affect protein expression or recognition by UP-R1, still the only available mAb specific for KIR2DL5. In this study, we build on those observations and demonstrate that phenotypic, functional, and structural diversity of KIR2DL5 depends, not only on copy number variation and variable transcription but also on allelic polymorphism targeting KIR2DL5 to either NK-cell surface expression or intracellular retention.

## Materials and Methods

### KIR2DL5–FLAG Constructs, Cell Lines Culture, and Transfection

A KIR2DL5A*001 cDNA clone with a FLAG epitope (DYKDDDDK) inserted between the leader peptide and the D0 Ig-like domain (6) was used to generate, by site-directed mutagenesis, three additional constructs bearing each of the missense mutations that distinguish the KIR2DL5A*001 and *005 primary structures—constructs N152D (152 AAT → GAT, asparagine to aspartate), G174S (GGC → AGC, glycine to serine), and KIR2DL5A*005 (both changes). Plasmids were purified using the EndoFree Plasmid Maxi and Midi Kits (Qiagen, Valencia, CA, USA) and sequenced with the universal primer SP6 and the internal primer R5e61 (5′-gttttggagcttggttcag-3′). Only error-free clones were used for transfection. Transfection experiments were replicated using up to five different plasmid batches per construct to control for random variations in expression attributable to DNA quality.

Human embryonic kidney (HEK)-293T cells were cultured in DMEM (Corning Inc., Corning, NY, USA) supplemented with 2 mM l-glutamine, 1% penicillin–streptomycin, and 10% FBS (Gibco Life Technologies, Carlsbad, CA, USA), and transiently co-transfected by the calcium phosphate method with 5 µg of each *KIR2DL5* construct and 0.1 µg of pEGFP-N1 vector (Clontech Laboratories, Palo Alto, CA, USA). Jurkat cells were cultured in RPMI-1640 Glutamax (Sigma-Aldrich, St. Louis, MO, USA) supplemented with 10% FBS and 1% penicillin–streptomycin, and transiently transfected with 2 µg of each *KIR2DL5* construct along with 0.1 µg of pEGFP-N1, using Solution V and the X-01 program of a Nucleofector I apparatus (Amaxa Biosystems, Cologne, Germany).

### Western Blot

Forty-eight hours after transfection, 2 × 10^5^ HEK-293T cells were lysed in 1% Non-idet P-40 Substitute (Sigma-Aldrich, St. Louis, MO, USA), 20 mM Tris–HCl, pH 8, 137 mM NaCl, 2 mM Na_2_EDTA lysis buffer containing 1% protease inhibitor cocktail (Roche Diagnostics GmbH, Mannheim, Germany). Proteins were reduced and denatured in 5× Laemmli buffer, run in 10% SDS-PAGE, and blotted to nitrocellulose membranes (iBlot Gel Transfer Stacks Nitrocellulose, Mini, Novex Life Technologies, Grand Island, NY, USA), which were treated for 1 h with blocking buffer (Li-Cor Bioscience, Lincoln, NE, USA). KIR2DL5–FLAG protein bands were detected with 1:1,000 dilution of anti-FLAG mAb M2 (Sigma-Aldrich, St. Louis, MO, USA), and 1:5,000 secondary antibody directed against mouse IgG (IRDye800, Li-Cor Bioscience). Beta-actin was detected, as a positive control, with 1:2,000 rabbit anti-human β-actin antibody (Abcam, Cambridge, UK) and the secondary reagent IRDye700 (Li-Cor Bioscience). Signals were detected with the Odyssey Infrared Imaging System (Li-Cor Bioscience). For N-deglycosylation, 400 µg of total cell lysates were denatured for 10 min at 100°C and treated with 2.5 U of Peptide-N-Glycosidase F (PNGase F, New England Biolabs, Ipswich, MA, USA) for 5 h at 37°C before Western blotting.

### Flow Cytometry

PBMCs or IL-2-expanded NK cells from healthy donors of known *KIR* genotype were incubated at 4°C with the following antibodies: APC anti-CD56 (eBioscience, Inc., San Diego, CA, USA), PE/Cy7 anti-CD3 (Biolegend, San Diego, CA, USA), and either PE anti-KIR2DL5 (UP-R1, Biolegend) or PE anti-KIR2DL2/L3/S2 (DX27, Miltenyi Biotec GmbH, Bergisch Gladbach, Germany). Isotype-matched negative controls were PE IgG1 (clone MOPC-21, Sigma-Aldrich) and PE IgG2a (clone S43.10, Miltenyi). Flow cytometry analysis was performed in MACSQuant Analyzer (Miltenyi) using MACSQuantify software. Percentages of KIR2DL5-positive cells were determined after gating on the CD3^−^CD56^+^ NK-cell population. The genotype of each donor was assessed using previously published polymerase chain reaction primers (14) targeting specific polymorphisms in the *KIR2DL5* coding and promoter regions; the latter primers also enabled to rule out potential confounding presence of transcribed *KIR2DL5B* alleles. This study was carried out in accordance with the recommendations of the Ethical Committee of Clinical Research of *Instituto de Investigación Sanitaria Puerta de Hierro*, which approved the protocol (270910-258). All participating volunteers provided written informed consent in accordance with the Declaration of Helsinki.

Primary mAbs used for flow cytometry of *KIR2DL5*-transfectants were anti-FLAG mAb M2 (Sigma-Aldrich), KIR2DL5-specific mAb UP-R1 (6), and mouse IgG1 isotype-matched negative control MOPC-21 (Sigma-Aldrich). PE-conjugated goat anti-mouse IgG + IgM, F(ab′)_2_ fragments (Jackson ImmunoResearch, West Grove, PA, USA) were used as secondary Ab. Jurkat and HEK-293T cells were stained 24 and 48 h post-transfection, respectively, and transient surface expression of KIR2DL5–FLAG was determined in Epics XL (Coulter Electronics, Hialeah, FL, USA) or MACSQuant (Miltenyi) flow cytometers, using Expo32 and MACSQuantify software, respectively. EGFP-positive cells ranged ~8–12% (Jurkat) and 30–40% (HEK-293T). Mean fluorescence intensities (MFIs) of different KIR2DL5 constructs, calculated after gating on EGFP^+^ cells, were compared using the paired Student’s *t*-test. To assess the effect of KIR2DL5 polymorphisms on recognition by mAb UP-R1, we calculated in each experiment the ratio of the MFIs obtained with KIR2DL5A*001 and each transfectant using M2 (anti-FLAG) and UP-R1, and ratios obtained for each mAb were compared using the non-parametric Wilcoxon signed-rank test.

### Confocal Microscopy

Forty-eight hours after transfection, HEK-293T cells were stained with anti-FLAG M2 mAb, fixed in PBS with 4% paraformaldehyde, and incubated with Alexa546-conjugated anti-mouse IgG (Invitrogen, Thermo Fisher Scientific, Waltham, MA, USA) to detect KIR2DL5–FLAG molecules on the surface. Then, cells were treated with 0.3% Triton X-100, and re-incubated with anti-FLAG M2 mAb, followed by Alexa448-labeled anti-mouse IgG (Invitrogen), to detect intracellular KIR2DL5 molecules. After this staining strategy, based on Ref. (21), cells were visualized on poly-l-lysine-coated glass-bottom dishes on a confocal laser-scanning microscope (TCS SP5, Leica Microsystems CMS GmbH, Mannheim, Germany) using Argon (488 nm) and Helium-Neon (543 nm) lasers and a 20×/0.5 lens. Images were acquired using the LAS AF SP5 software (Leica).

### KIR2DL5A Structure Model

We based on the KIR2DL1 X-ray structure (22) (PDB accession code: 1NKR) to predict the structures of the D2 Ig-like domains of KIR2DL5A*001 and *005, using the SWISS-MODEL automated protein structure homology-modeling system (23), accessible *via* the ExPASy web server.^1^ Quality of each model was assessed with the QMEAN *Z*-score (24), which describes the likelihood that a model quality is comparable to high-resolution experimental structures.^2^ Structures were visualized and edited using the PyMOL Molecular Graphics System (version 1.7.4, Schrödinger, LLC).

## Results

### Allelic Polymorphism Affects KIR2DL5A Detection on the NK-Cell Surface by Flow Cytometry

**Figure 1:**
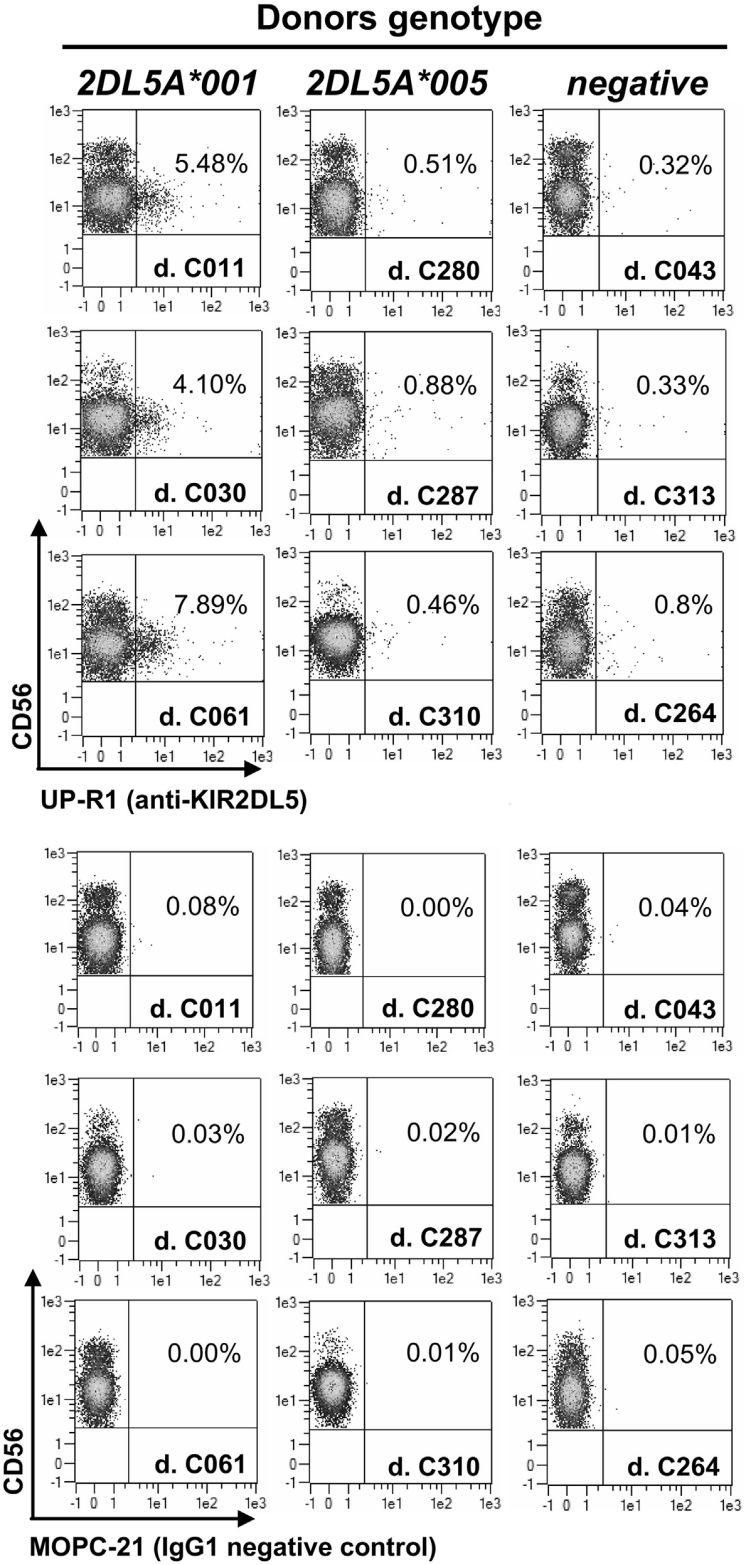
**Flow cytometry profiles of NK cells from donors with different *KIR2DL5A* genotypes**. Unmanipulated NK cells from peripheral blood of three donors expressing KIR2DL5A*001 (left, positive control), three carrying KIR2DL5A*005 (middle), and three lacking a transcribed *KIR2DL5A* or *KIR2DL5B* gene (right, negative control).

Flow cytometry assays of PBMCs from donors with a KIR2DL5A*001 allele identify discrete subpopulations of NK cells reacting with the only available anti-KIR2DL5 mAb, UP-R1 (average of positive NK cells in 11 donors ± SD: 5.18 ± 2.15%; range: 1.71–7.89%), consistently with the clonal expression pattern reported for the receptor (4, 6). In contrast, much fewer UP-R1-positive NK cells are seen in donors carrying allele KIR2DL5A*005 (0.50 ± 0.24%; range: 0.23–0.93%; *n* = 9; *p* = 0.0001), their proportions being not significantly different from the background seen in donors lacking a transcribed *KIR2DL5* gene (0.33 ± 0.20%; range: 0.15–0.78%; *n* = 11). In fact, the flow cytometry plots of donors pertaining to the latter two categories are hardly distinguishable on an individual basis (Figure 1), indicating that NK cells of KIR2DL5A*005 subjects are, for some reason, unreactive with UP-R1 in these assays.

**Figure 2:**
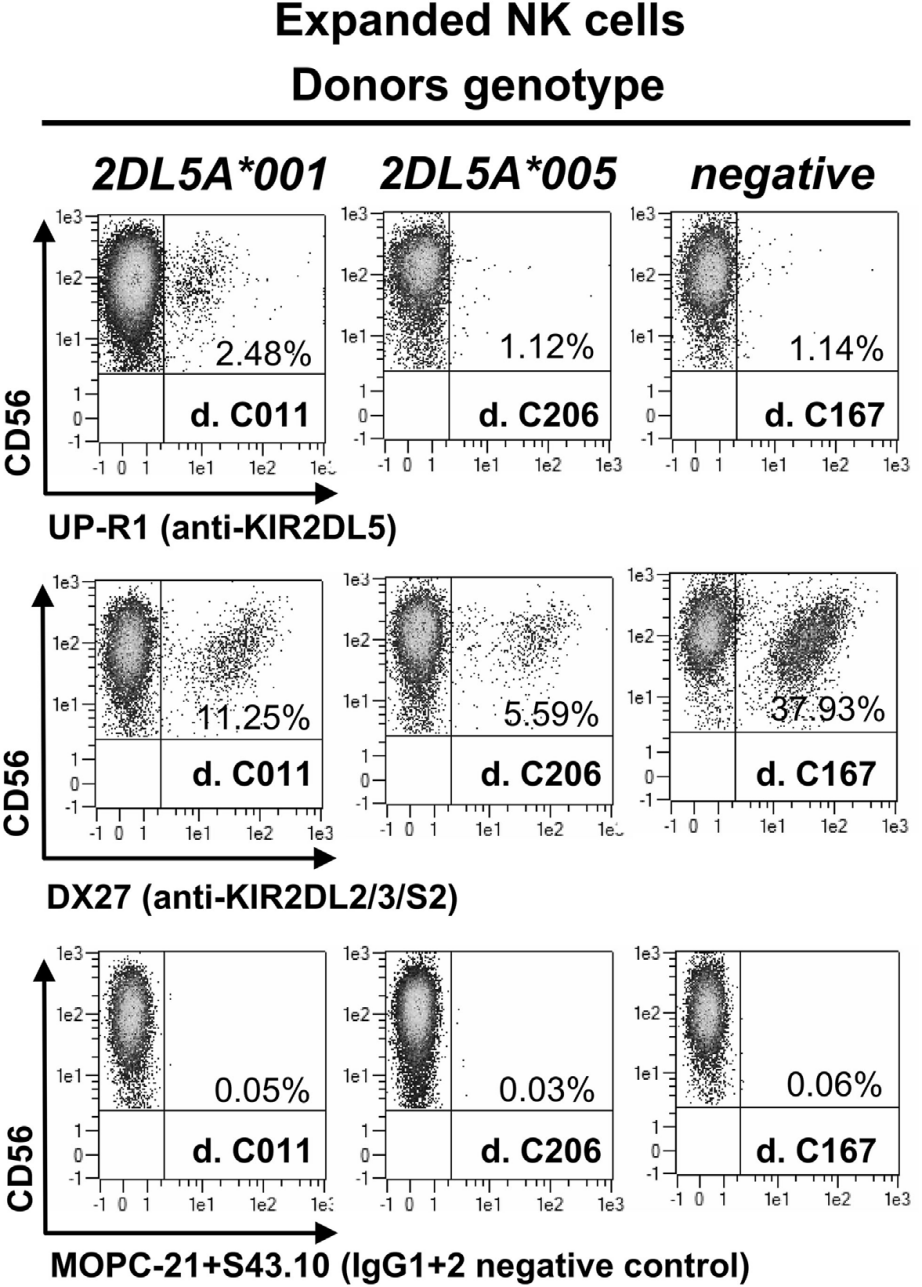
**NK cells expanded *in vitro* with IL-2, isolated from three donors, each of them representing a different *KIR2DL5A* genotype**. MOPC21 (IgG1) and S43.10 (IgG2) are isotype-matched negative controls for the anti-KIR2DL5 (UP-R1) and anti-KIR2DL2/L3/S2 (DX27) mAbs.

KIR2DL5A*001-positive NK cells expanded *in vitro* express higher levels of the receptor on the surface than resting cells, as it happens with other KIR (Figure 2). In contrast, NK cells expanded from individuals carrying KIR2DL5A*005 appear to upregulate no product detectable with UP-R1, remaining indistinguishable from those of a *KIR2DL5A*-negative donor (Figure 2). This argues against the possibility of a KIR2DL5A*005 product being expressed at low levels on the surface of freshly isolated NK cells. Given that KIR2DL5A*005 mRNA is as readily detected as that of the common allele KIR2DL5A*001 (14), these results suggest that the putative KIR2DL5A*005 product is not transported to the cell surface or fails to react with mAb UP-R1.

### Predictive Analysis of Posttranslational Modifications, Subcellular Location, and Folding Motifs in KIR2DL5A Alleles

**Figure 3:**
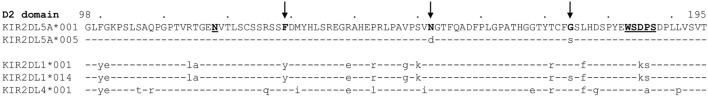
Comparison of the primary structures of the KIR2DL5A*001 and *005 D2 Ig-like domains, along with those of *KIR2DL1* alleles *001 and *014, and KIR2DL4*001. Arrows mark *KIR2DL5A* polymorphic positions 152 and 174, and Phe-129, predictably confronted to Gly/Ser-174 in the receptor architecture. Other residues mentioned in the text are underlined. Amino acid numbering is based on the mature *KIR2DL5A* polypeptide (the KIR2DL1 N-terminal Ig-like domain, not represented, is five amino acids longer than those of killer-cell Ig-like receptors 2DL4 and 2DL5).

Because of the crucial role of protein posttranslational modifications on several cellular processes, including maturation and protein location, we analyzed whether potential KIR2DL5A posttranslational processing could be predictably affected by its allelic polymorphism. The two substitutions that distinguish the primary structures of KIR2DL5A*001 and *005 are located in the D2 Ig-like domain and involve amino acids with side chains susceptible to glycosylation—asparagine/aspartate-152 and glycine/serine-174. Therefore, we performed a predictive search of possible O- and N-glycosylation motifs in the sequences of their D2 domains. None of the predicted O-glycosylation sites involves KIR2DL5 polymorphic positions (not shown). In contrast, substitution of asparagine-152 by aspartate in KIR2DL5A*005 destroys an N-glycosylation site (Figure 3) highly conserved in all KIR (25), whose loss might affect KIR2DL5A*005 surface expression.

We also explored possible presence in the KIR2DL5A*005 primary structure of sequence motifs that could affect its subcellular location or folding. However, we found no signals for endoplasmic reticulum (ER) retention, such as di-basic (i.e., arginine/lysine) motifs, or variants of an H/KDEL sequence seen in ER-resident proteins (26). Furthermore, KIR2DL5A*005, like allele *001, has intact WSXPS motifs in each of its Ig-like domains, mutations of which have been reported to hamper correct folding and surface expression of the KIR3DL1*004 allele (27), as well as cytokine receptors (28).

### Allele KIR2DL5A*005 Is Weakly Expressed on the Surface of Transfected Cells and Is Poorly Recognized by KIR2DL5-Specific mAb UP-R1

**Figure 4:**
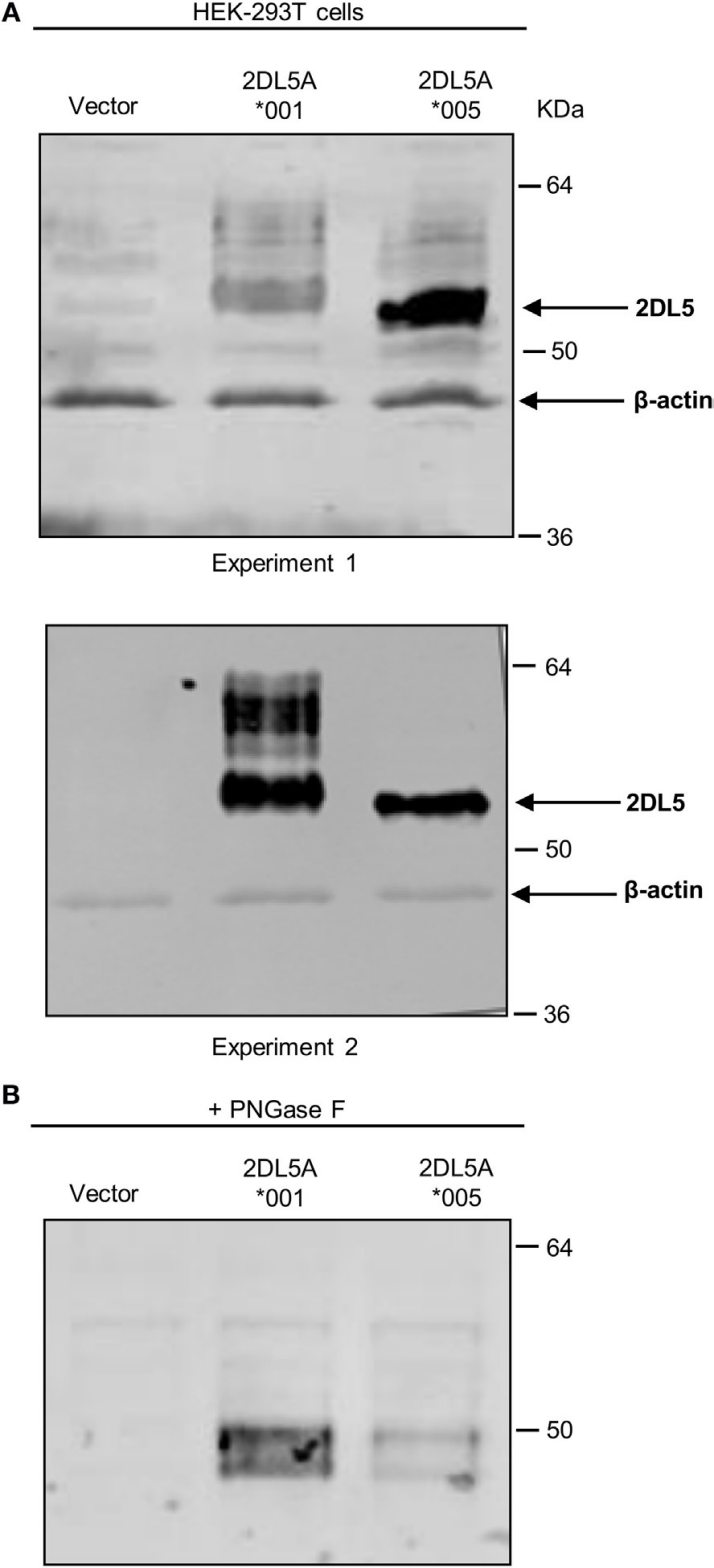
**Western blot analysis of KIR2DL5A expression in transfected human embryonic kidney (HEK)-293T cells**. **(A)** KIR2DL5A was immunoprecipitated from lysates of HEK-293T cells expressing KIR2DL5A*001 or KIR2DL5A*005 using anti-FLAG mAb. Beta-actin enables monitoring the amount of cell lysate loaded on the gel. Two duplicate independent experiments are shown. **(B)** Cell lysates were digested with Peptide-N-Glycosidase F before Western blot with anti-FLAG.

To reliably track the expression of KIR2DL5A*001 and *005, we tagged cDNA constructs of these alleles with a FLAG epitope which should enable us to detect their products independently of UP-R1 recognition. Such constructs were transfected into HEK-293T cells, and expression was verified by western blot with an anti-FLAG mAb. As shown in Figure 4A, specific bands of 50–60 kDa were seen in both KIR2DL5A*001 and *005-transfected cells, in comparison with mock-transfected HEK-293T. Differences in the relative mobility of KIR2DL5A*001 and *005 were observed, and they are attributable to their differential N-glycosylation, since they disappear after treatment of the cell lysates with PNGase F (Figure 4B). Also, due to variable transfection efficacy, the relative amounts of each synthesized protein varied between experiments (e.g., experiments 1 and 2 of Figure 4A). To compensate for such variations, transfections were replicated six times using different plasmid batches, EGFP was co-transfected in all experiments, and KIR2DL5 expression was evaluated on EGFP^+^ cells.

**Figure 5:**
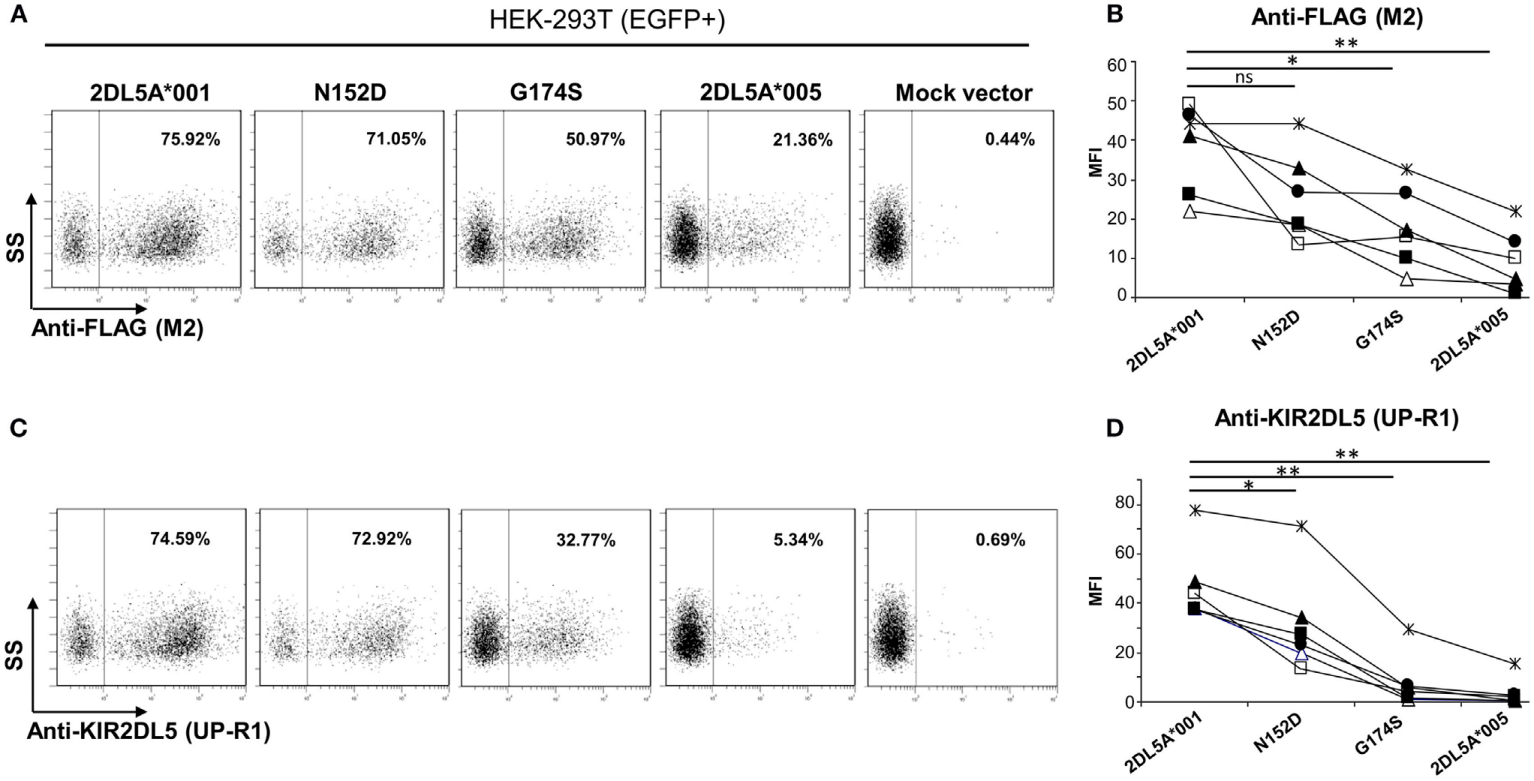
**Flow cytometry analysis of surface KIR2DL5A expression on transfected human embryonic kidney-293T cells, using anti-FLAG (A,B) and anti-KIR2DL5 mAbs (C,D), after gating on EGFP^+^ cells**. Dot plots in panels **(A,C)** depict one representative experiment of six analyzed in graphs **(B,D)**; different symbols are used in these graphs for each experiment. To compare KIR2DL5 expression levels and compensate for variations in transfection efficacy, the mean fluorescence intensity achieved with M2 **(B)** and UP-R1 **(D)** mAbs was measured on cells expressing EGFP, cotransfected along with KIR2DL5 in each experiment. *p < 0.01, **p < 0.001; ns, not significant; SS, side scatter.

Flow cytometry analysis of *KIR2DL5A*-transfected HEK-293T cells with anti-FLAG mAb revealed discrete subpopulations of cells showing specific surface expression of both KIR2DL5A*001 and *005 (Figures 5A,B). Density of the former molecule was, however, consistently and significantly higher than that of the minor allele KIR2DL5A*005 (average MFI of six experiments ± SD: 38 ± 11 vs. 9 ± 8; *p* = 0.0004). Similarly, flow cytometry with the KIR2DL5-specific mAb UP-R1 showed that KIR2DL5A*001-transfected cells are stained more intensely than those expressing KIR2DL5A*005 (MFI 47 ± 16 vs. 4 ± 6; *p* = 0.0001, Figures 5C,D). These results roughly replicate the observed natural behavior of these alleles, except for the fact that KIR2DL5A*005 is only detectable on transfected cells. Furthermore, comparison of the staining profiles obtained with anti-FLAG M2 and anti-KIR2DL5 UP-R1 mAbs showed that, despite following similar trends, differences in apparent expression levels of KIR2DL5A*001 and *005 were somewhat more pronounced when cells were stained with UP-R1 mAb (Figure 5). For instance, the MFI values of KIR2DL5*001 cells were 5.1- to 75.0-fold higher than those of *005 ones when measured with UP-R1, but only 2.0- to 21.6-fold higher when assessed with the anti-FLAG mAb, the difference being statistically significant (*p* < 0.05) and consistently seen in every experiment. This behavior suggests that KIR2DL5A*005 detection by UP-R1 is limited, not only by lower expression of the molecule but also by poorer recognition by the antibody.

### Differential Contribution of the G174S and N152D Polymorphisms to the KIR2DL5A*005 Phenotype

To assess the relative contribution of the KIR2DL5A*005 polymorphisms to reduced surface expression and UP-R1 mAb recognition of this allele, we introduced separately the G174S and N152D mutations into the KIR2DL5A*001 coding region, and analyzed expression on transfected HEK-293T cells as above, using anti-FLAG and anti-KIR2DL5 mAbs.

Examination of the mutants flow cytometry profiles with the anti-FLAG antibody (Figures 5A,B) showed that substitution of aspartate for asparagine-152 induced only a modest effect on KIR2DL5A expression levels (MFI: 26 ± 11 vs. 38 ± 11 in KIR2DL5A*001, *p* = 0.0695). In contrast, the serine for glycine-174 mutant displayed a significant reduction in surface expression in comparison with the wild-type allele (MFI: 18 ± 10 vs. 38 ± 11, *p* = 0.0013). Western blot analyses of the two mutants revealed comparable levels of KIR2DL5 protein synthesis (not shown).

Analysis of expression with the KIR2DL5-specific mAb UP-R1 replicated the results obtained with the anti-FLAG antibody, except for the facts that both mutants differed significantly from KIR2DL5A*001 (Figure 5D); and that, as it happened in the comparison of KIR2DL5A*001 and *005, the decrease in recognition of the G174S mutant was more pronounced with UP-R1 than with the anti-FLAG antibody (2DL5A*001/G174S MFI ratios: UP-R1, 2.7–29.5; anti-FLAG, 1.4–4.6, *p* < 0.05). In fact, G174S mutants showed an average MFI similar to that of the low-expressed allele KIR2DL5A*005 (8 ± 11 vs. 4 ± 6), supporting that serine-174 hampers recognition by the KIR2DL5-specific antibody besides reducing expression levels.

**Figure 6:**
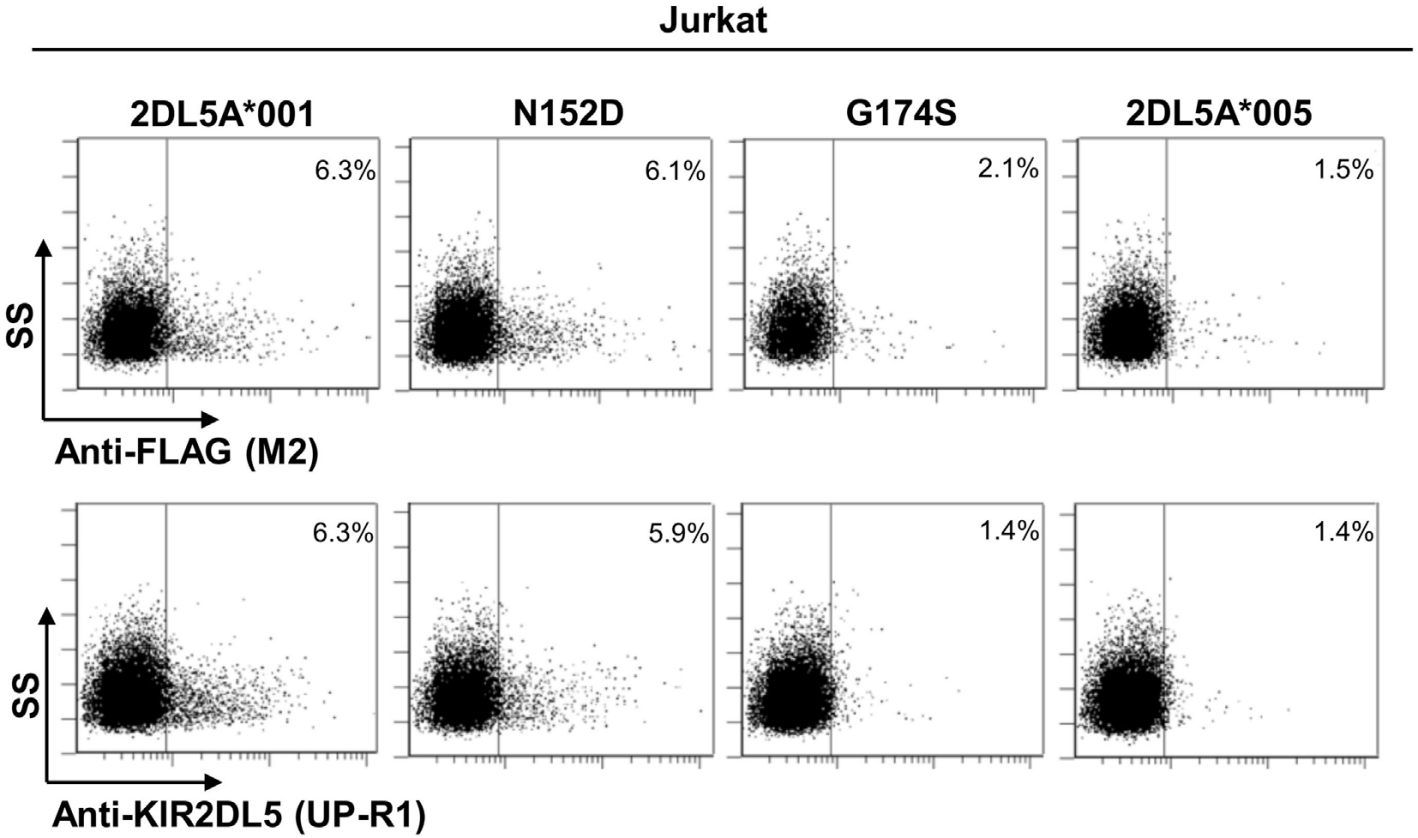
**Flow cytometry analysis of surface KIR2DL5A expression on nucleofected Jurkat cells, using anti-FLAG and anti-KIR2DL5 mAbs**.

To further validate our findings, we replicated the flow cytometry analysis of each *KIR2DL5* construct after nucleofection into Jurkat, a T-cell line previously used in studies of KIR expression (27, 29, 30). In line with the less effective transfection of Jurkat, expression levels of all *KIR2DL5* constructs emulated more closely those seen in NK cells of KIR2DL5A*001- and *005-positive subjects, being 2- to 10-fold lower than those obtained with HEK-293T cells. Despite this difference, the relative expression of each construct in comparison with KIR2DL5A*001, determined with either UP-R1 or anti-FLAG mAbs, roughly reproduced that previously obtained in HEK-293T cells; i.e., percentages of cells with appreciable receptor density on the surface were conspicuously lower for KIR2DL5A*005 and the G174S mutant than for KIR2DL5A*001, whereas the N152D mutation had only a minor effect on KIR2DL5 expression and recognition by UP-R1 (Figure 6).

### Allele KIR2DL5A*005 Is Retained Intracellularly Due to a Serine for Glycine Substitution in the D2 Ig-Like Domain

Our previous results show that KIR2DL5A*005 translation is not followed by efficacious expression on the cell surface. To assess whether the KIR2DL5A*005 product is retained intracellularly, we performed confocal microscopy experiments of HEK-293T cells transfected with FLAG-tagged constructs of alleles KIR2DL5A*001 and *005, and with the above described intermediate mutants N152D and G174S.

**Figure 7:**
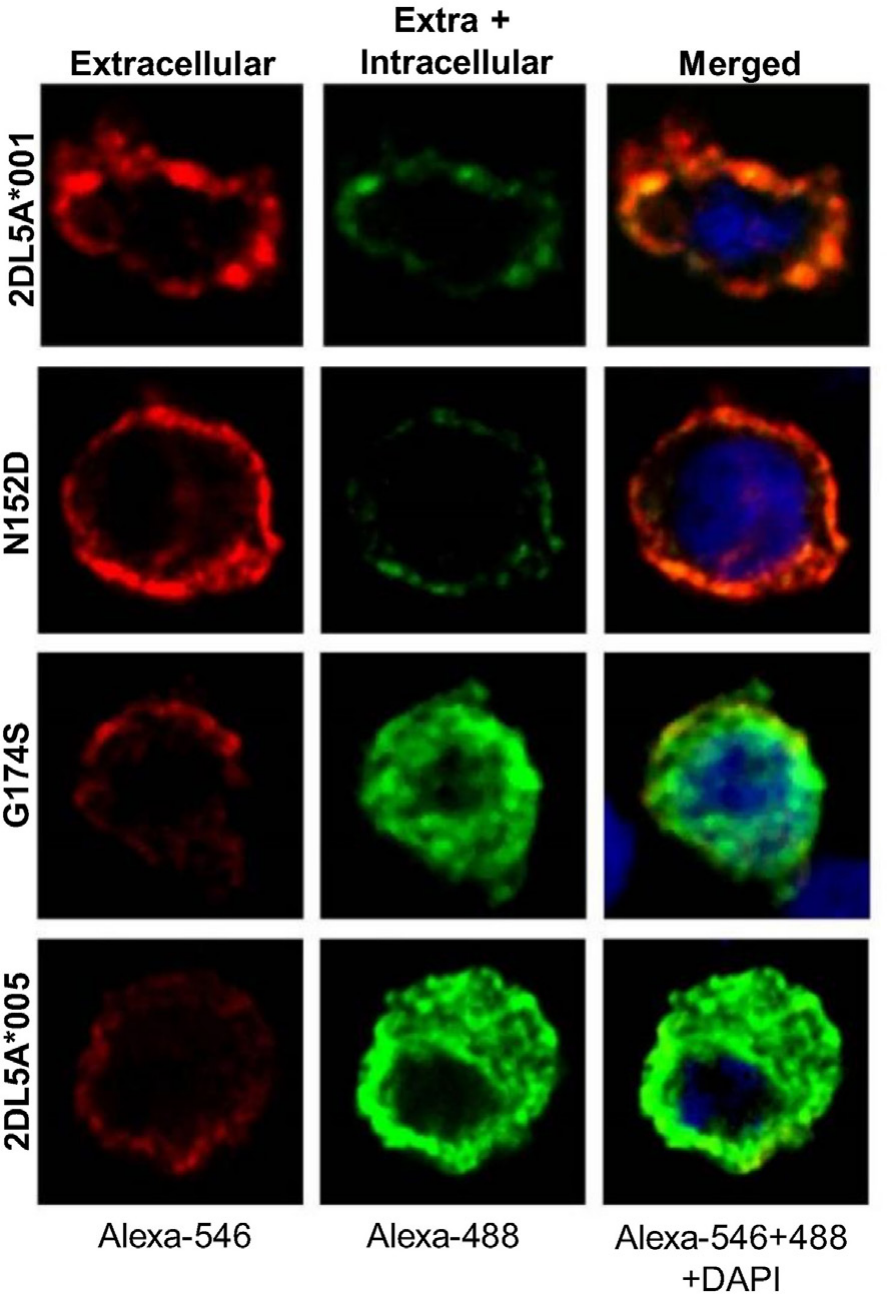
**Confocal microscopy analysis of surface and intracellular KIR2DL5A expression in transfected human embryonic kidney-293T cells, using an anti-FLAG mAb**. Cells were stained before (left) and after (middle) membrane permeabilization, using secondary reagents labeled with different dyes in each step.

As shown in Figure 7, remarkable differences were seen in the cellular distribution of the four transfected constructs, as assessed with an anti-FLAG mAb. In cells expressing KIR2DL5A*001 or the N152D mutant, fluorescence was localized on the plasma membrane, without appreciable intracellular staining. In contrast, KIR2DL5A*005, as well as mutant G174S, were predominantly detected inside transfected cells, despite a small amount of molecules colocalizing with the plasma membrane, in agreement with the flow cytometry results. Unfortunately, KIR2DL5-specific mAb UP-R1 behaved poorly after cell fixation–permeabilization, which precluded subsequent studies of intracellular staining with this mAb to track KIR2DL5A*005 localization in NK cells expressing naturally this molecule.

**Figure 8:**
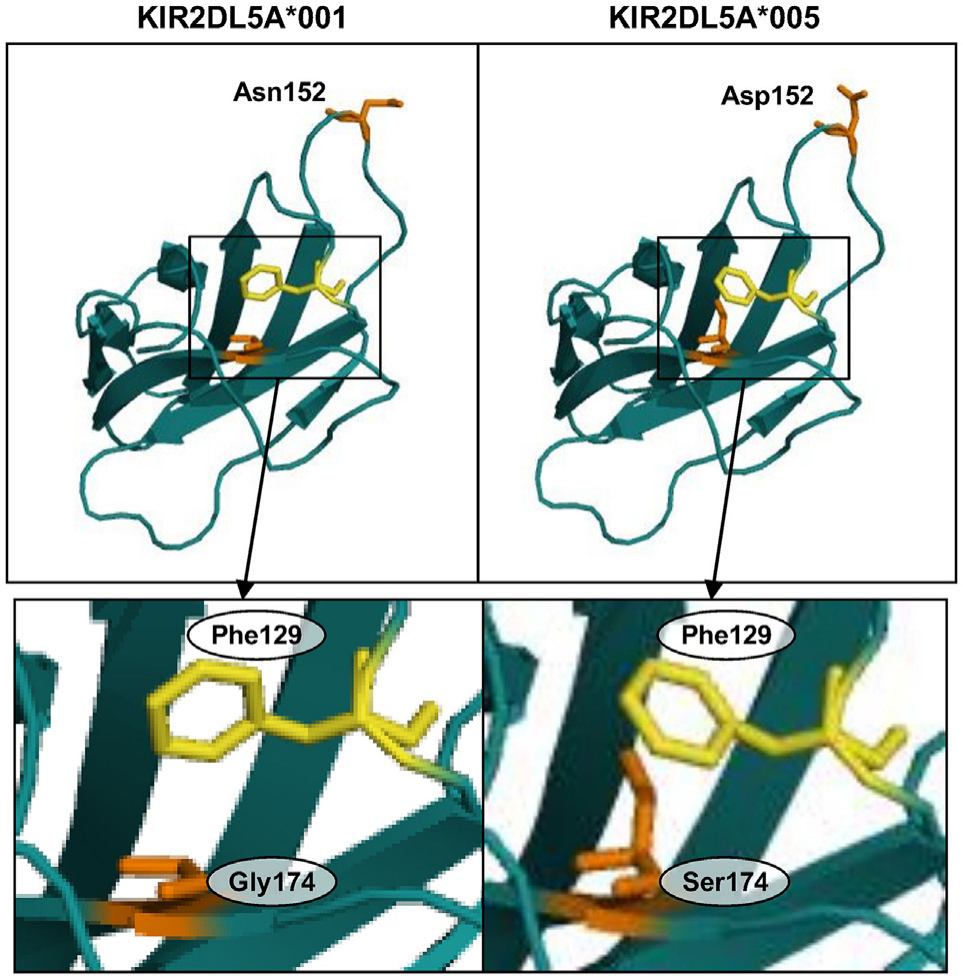
Predicted structure of the D2 Ig-like domains of KIR2DL5 alleles *001 and *005. Insets highlight displacement of phenylalanine-129 in the KIR2DL5A*005 model due to a spatial constraint posed by the serine-174 side chain.

To gain insight on the mechanism by which a serine for glycine-174 change in the D2 Ig-like domain could hinder KIR2DL5A surface expression, we analyzed this substitution in a predicted three-dimensional model of the receptor. To that end, and since the crystallographic structure of KIR2DL5 has not yet been solved, we modeled its D2 domain on the structure of KIR2DL1 (31), which, among all KIR, shares with KIR2DL5 the highest amino acid sequence identity in that region (86.73% with no gaps). QMEAN6 *Z*-scores, estimating the absolute quality of the KIR2DL5*001 and *005 models, were 0.25 and −0.43 SDs, respectively, from the KIR2DL1 experimental structure. KIR2DL5 residue 152 (Asn/Asp) is exposed on the protein surface (segment connecting β-strands D and E), while amino acid 174 (Gly/Ser) is partially hidden inside the receptor architecture (strand F), confronted with phenylalanine-129 (loop 5, near strand C). Replacement of glycine-174 in the KIR2DL5A*001 model by serine of KIR2DL5A*005 induces displacement of phenylalanine-129 (Figure 8), as a consequence of the bigger size of the serine hydroxymethyl group in comparison with the hydrogen side chain of glycine-174. Of note, a similar displacement of KIR2DL1 tyrosine-134 (paralogous of KIR2DL5 Phe-129, Figure 3), resulting in intracellular retention, has been attributed to an identical Gly-to-Ser substitution in allele KIR2DL1*014 residue 179 (32), paralogous of KIR2DL5 amino acid 174. KIR2DL5 substitutions G174S and N152D appear to affect no other bonds or interactions between surrounding amino acids, and we found no additional clues to the KIR2DL5A*005 behavior by modeling on KIR2DL4 (PDB accession code 3WYR, results not shown), receptor that, in contrast with KIR2DL1, shares with KIR2DL5 the D0–D2 Ig-like domain organization, but slightly lower percentage of identity in the primary structure.

## Discussion

The KIR2DL5 function and importance in human health are poorly understood. Studies on this KIR are complicated by its entangled genetics and expression profiles. Most conspicuous in this regard is frequent discrepancy between detection of the receptor by flow cytometry and simple forms of *KIR* genotyping that inform only of presence or absence of a *KIR2DL5* gene. Such contradicting results (i.e., no protein detectable by flow cytometry in a *KIR2DL5*^+^ individual) are partly explained by most *KIR2DL5B* alleles being epigenetically silenced, which can be predicted reliably by genotyping the -97 A/G dimorphism of the *KIR2DL5* promoter—only alleles with G (part of a RUNX-binding site) are transcribed (14). In this study, we have addressed another source of apparent discrepancy between KIR2DL5 phenotyping and basic genotyping—lack of a detectable product despite presence of a *KIR2DL5* allele with an intact RUNX site. We have shown here that this situation is regularly seen in KIR2DL5A*005, part of the common telomeric *KIR* motif 3DS1-2DL5A*005-2DS3*002-2DS1-3DL2 (7, 10, 11, 17). According to our results, such phenotype can be anticipated also for KIR2DL5B*0020106 and *00202, centromeric alleles governed by predictably functional promoters and encoding mature polypeptides identical to KIR2DL5A*005 (16).

Western blot, flow cytometry, and confocal microscopy studies of cells transfected with tagged *KIR2DL5A* constructs show existence of a KIR2DL5A*005-gene product that, in comparison with the common allele KIR2DL5A*001, scarcely reaches the plasma membrane and is mostly retained intracellularly. Comparison of the flow cytometry profiles of mAbs directed against the tag and the receptor also reveal that the only available KIR2DL5-specific mAb, UP-R1, binds to 2DL5A*005 with lower efficacy than to 2DL5A*001, suggesting that its epitope is somehow altered in the former allele. In consequence, we cannot exclude completely that KIR2DL5A*005 traces reach the NK-cell surface at levels undetectable by the mAb, as shown for another KIR retained intracellularly, 3DL1*004 (30). Against this possibility is that NK cells expanded from KIR2DL5A*005 subjects remain negative for UP-R1. We have also not ruled out that intracellular KIR2DL5 might exert some function. In this regard, 2DL4, the only other human KIR with D0–D2 domains, has been reported to signal from its preferential intracellular location (33).

The two amino acid changes in the membrane-proximal D2 Ig-like domain that distinguish KIR2DL5A*005 from allele *001 (and most other KIR) appear to have independent and different contributions to its phenotype, according to the behavior of cells transfected with the individual mutant sequences. Replacement of asparagine-152 by aspartate appears to have only a minor effect on expression and UP-R1 mAb recognition in spite of destroying an N-glycosylation site well conserved in KIR, while substitution of serine for glycine-174 is enough for inducing intracellular retention, reducing drastically surface expression and impairing mAb recognition.

The reason for serine-174 hampering KIR2DL5 expression is apparently subtle. Three-dimensional modeling only shows that the serine side chain forces, through steric hindrance, displacement of the phenylalanine-129 aromatic residue from its place in KIR2DL5A*001. It is possible that such displacement results in some disorganization of the D2 Ig-like domain architecture, hampering proper folding and favoring intracellular retention of the receptor, rather than trafficking to the cell surface. In support of this possibility is that a similar phenomenon has been reported for KIR2DL1, in which an identical polymorphism also associates with lack of surface expression (32). Such disorganization could also explain that a hardly exposed amino acid affects UP-R1 mAb recognition.

KIR2DL5 is, in many aspects, an intriguing molecule. Contrasting with most human KIR, it is seen in several primate species, feature shared by KIR2DL4. But, unlike the latter KIR, 2DL5 is not conserved in all humans; on the contrary, despite its gene being duplicated (trait specific of our species), nearly a half of human genomes lack both *KIR2DL5A* and *KIR2DL5B* (7). Furthermore, the two loci are represented by alleles known or expected to be expressed on the NK-cell surface (e.g., 2DL5A*001, 2DL5B*00602); and by ones that are not, either due to silenced transcription (e.g., 2DL5B*00601), to amino acid substitutions that induce intracellular retention (e.g., 2DL5A*005), or both (the common 2DL5B*002 alleles). The fact that surface and intracellularly expressed and also non-expressed *KIR2DL5* alleles are all maintained at significant frequencies in different human populations suggests that balancing selection favors persistence of functionally divergent KIR2DL5 allotypes that might help humans to survive to different selective pressures. Elucidation of such pressures and comprehension of other aspects of KIR2DL5 function await identification of the molecules recognized by the receptor.

In summary, we have shown that a common polymorphism in the *KIR2DL5* coding sequence targets the encoded receptor for intracellular expression, which, together with variations in its promoter, explains all apparent discrepancies between KIR2DL5 geno- and phenotyping. A major implication of our results is that analyses of NK-cell repertoire and genetic association studies aimed at exploring or understanding relationship of KIR2DL5 with different health conditions will only be meaningful if its allelic polymorphism and different expression profiles are taken into account.

## Author Contributions

EC designed and performed experiments, analyzed and interpreted data, and wrote the manuscript. EE designed and performed experiments, and analyzed and interpreted data. CV designed the study, directed research, and wrote the manuscript.

## Conflict of Interest Statement

The Immunogenetics and Histocompatibility group of *Instituto de Investigación Sanitaria Puerta de Hierro*, directed by CV, shares with Dr. Miguel López-Botet (*Universitat Pompeu Fabra*, Barcelona, Spain) rights to monoclonal antibody UP-R1 and has received license fees and royalties from companies selling the antibody. The authors declare that the research was conducted in the absence of any other commercial or financial relationships that could be construed as a potential conflict of interest.

## Footnotes

^1^http://swissmodel.expasy.org/.

^2^https://swissmodel.expasy.org/qmean/cgi/index.cgi?page=help.

## Abbreviations

HEK, human embryonic kidney; KIR, killer-cell Ig-like receptor.

## References

[1] Colonna M Samaridis J. Cloning of immunoglobulin-superfamily members associated with HLA-C and HLA-B recognition by human natural killer cells. Science (1995) 268(5209):405–8. 10.1126/science.7716543 7716543

[2] Wagtmann N Biassoni R Cantoni C Verdiani S Malnati MS Vitale M Molecular clones of the p58 NK cell receptor reveal immunoglobulin-related molecules with diversity in both the extra- and intracellular domains. Immunity (1995) 2(5):439–49. 10.1016/1074-7613(95)90025-X 7749980

[3] Vilches C Parham P. KIR: diverse, rapidly evolving receptors of innate and adaptive immunity. Annu Rev Immunol (2002) 20:217–51. 10.1146/annurev.immunol.20.092501.134942 11861603

[4] Vilches C Rajalingam R Uhrberg M Gardiner CM Young NT Parham P. KIR2DL5, a novel killer-cell receptor with a D0-D2 configuration of Ig-like domains. J Immunol (2000) 164(11):5797–804. 10.4049/jimmunol.164.11.5797 10820258

[5] Wilson MJ Torkar M Haude A Milne S Jones T Sheer D Plasticity in the organization and sequences of human KIR/ILT gene families. Proc Natl Acad Sci U S A (2000) 97(9):4778–83. 10.1073/pnas.080588597 10781084 PMC18309

[6] Estefanía E Flores R Gómez-Lozano NG Aguilar H López-Botet ML Vilches C. Human KIR2DL5 is an inhibitory receptor expressed on the surface of NK and T lymphocyte subsets. J Immunol (2007) 178(7):4402–10. 10.4049/jimmunol.178.7.4402 17371997

[7] Cisneros E Moraru M Gómez-Lozano N López-Botet M Vilches C KIR2DL5: an orphan inhibitory receptor displaying complex patterns of polymorphism and expression. Front Immunol (2012) 3:289 10.3389/fimmu.2012.00289 23060877 PMC3443818

[8] Vilches C Gardiner CM Parham P Gene structure and promoter variation of expressed and non-expressed variants of the KIR2DL5 gene. J Immunol (2000) 165:6416–21. 10.4049/jimmunol.165.11.6416 11086080

[9] Gómez-Lozano N Gardiner CM Parham P Vilches C. Some human KIR haplotypes contain two KIR2DL5 genes: KIR2DL5A and KIR2DL5B. Immunogenetics (2002) 54(5):314–9. 10.1007/s00251-002-0476-2 12185535

[10] Ordóñez D Meenagh A ómez-Lozano NG Castaño J Middleton D Vilches C. Duplication, mutation and recombination of the human orphan gene KIR2DS3 contribute to the diversity of KIR haplotypes. Genes Immun (2008) 9(5):431–7. 10.1038/gene.2008.34 18480828

[11] Vierra-Green C Roe D Hou L Hurley CK Rajalingam R Reed E Allele-level haplotype frequencies and pairwise linkage disequilibrium for 14 KIR loci in 506 European-American individuals. PLoS One (2012) 7(11):e47491. 10.1371/journal.pone.0047491 23139747 PMC3489906

[12] Jiang W Johnson C Jayaraman J Simecek N Noble J Moffatt MF Copy number variation leads to considerable diversity for B but not A haplotypes of the human KIR genes encoding NK cell receptors. Genome Res (2012) 22(10):1845–54. 10.1101/gr.137976.112 22948769 PMC3460180

[13] Robinson J Mistry K McWilliam H Lopez R Marsh SG IPD – the immuno polymorphism database. Nucleic Acids Res (2010) 38:D863–9. 10.1093/nar/gkp879 19875415 PMC2808958

[14] Gómez-Lozano N Trompeter HI de Pablo R Estefanía E Uhrberg M Vilches C. Epigenetic silencing of potentially functional KIR2DL5 alleles: implications for the acquisition of KIR repertoires by NK cells. Eur J Immunol (2007) 37(7):1954–65. 10.1002/eji.200737277 17557377

[15] Du Z Sharma SK Spellman S Reed EF Rajalingam R. KIR2DL5 alleles mark certain combination of activating KIR genes. Genes Immun (2008) 9(5):470–80. 10.1038/gene.2008.39 18509341

[16] Hou L Chen M Jiang B Wu D Ng J Hurley CK. Thirty allele-level haplotypes centered around KIR2DL5 define the diversity in an African American population. Immunogenetics (2010) 62(8):491–8. 10.1007/s00251-010-0458-8 20585770 PMC3485642

[17] Hou L Chen M Ng J Hurley CK. Conserved KIR allele-level haplotypes are altered by microvariation in individuals with European ancestry. Genes Immun (2012) 13(1):47–58. 10.1038/gene.2011.52 21796155 PMC3536055

[18] Trompeter HI Gómez-Lozano N Santourlidis S Eisermann B Wernet P Vilches C Three structurally and functionally divergent kinds of promoters regulate expression of clonally distributed killer cell Ig-like receptors (KIR), of KIR2DL4, and of KIR3DL3. J Immunol (2005) 174(7):4135–43. 10.4049/jimmunol.174.7.4135 15778373

[19] Gómez-Lozano N Estefanía E Williams F Halfpenny I Middleton D Solís R The silent KIR3DP1 gene (CD158c) is transcribed and might encode a secreted receptor in a minority of humans, in whom the KIR3DP1, KIR2DL4 and KIR3DL1/KIR3DS1 genes are duplicated. Eur J Immunol (2005) 35(1):16–24. 10.1002/eji.200425493 15580659

[20] Mulrooney TJ Hou L Steiner NK Chen M Belle I Ng J Promoter variants of KIR2DL5 add to diversity and may impact gene expression. Immunogenetics (2008) 60(6):287–94. 10.1007/s00251-008-0273-7 18461314

[21] Sierra-Filardi E Estecha A Samaniego R Fernandez-Ruiz E Colmenares M Sanchez-Mateos P Epitope mapping on the dendritic cell-specific ICAM-3-grabbing non-integrin (DC-SIGN) pathogen-attachment factor. Mol Immunol (2010) 47(4):840–8. 10.1016/j.molimm.2009.09.036 19879650

[22] Fan QR Garboczi DN Winter CC Wagtmann N Long EO Wiley DC. Direct binding of a soluble natural killer cell inhibitory receptor to a soluble human leukocyte antigen-Cw4 class I major histocompatibility complex molecule. Proc Natl Acad Sci U S A (1996) 93(14):7178–83. 10.1073/pnas.93.14.7178 8692965 PMC38956

[23] Biasini M Bienert S Waterhouse A Arnold K Studer G Schmidt T SWISS-MODEL: modelling protein tertiary and quaternary structure using evolutionary information. Nucleic Acids Res (2014) 42:W252–8. 10.1093/nar/gku340 24782522 PMC4086089

[24] Benkert P Biasini M Schwede T. Toward the estimation of the absolute quality of individual protein structure models. Bioinformatics (2011) 27(3):343–50. 10.1093/bioinformatics/btq662 21134891 PMC3031035

[25] Garcia CA Robinson J Guethlein LA Parham P Madrigal JA Marsh SG Human KIR sequences 2003. Immunogenetics (2003) 55(4):227–39. 10.1007/s00251-003-0572-y 12838379

[26] Teasdale RD Jackson MR. Signal-mediated sorting of membrane proteins between the endoplasmic reticulum and the Golgi apparatus. Annu Rev Cell Dev Biol (1996) 12:27–54. 10.1146/annurev.cellbio.12.1.27 8970721

[27] Pando MJ Gardiner CM Gleimer M McQueen KL Parham P. The protein made from a common allele of KIR3DL1 (3DL1*004) is poorly expressed at cell surfaces due to substitution at positions 86 in Ig domain 0 and 182 in Ig domain 1. J Immunol (2003) 171(12):6640–9. 10.4049/jimmunol.171.12.6640 14662867

[28] Doshi PD DiPersio JF. Three conserved motifs in the extracellular domain of the human granulocyte-macrophage colony-stimulating factor receptor subunit are essential for ligand binding and surface expression. Blood (1994) 84(8):2539–53. 7919372

[29] VandenBussche CJ Mulrooney TJ Frazier WR Dakshanamurthy S Hurley CK. Dramatically reduced surface expression of NK cell receptor KIR2DS3 is attributed to multiple residues throughout the molecule. Genes Immun (2009) 10(2):162–73. 10.1038/gene.2008.91 19005473 PMC3487464

[30] Taner SB Pando MJ Roberts A Schellekens J Marsh SG Malmberg KJ Interactions of NK cell receptor KIR3DL1*004 with chaperones and conformation-specific antibody reveal a functional folded state as well as predominant intracellular retention. J Immunol (2011) 186(1):62–72. 10.4049/jimmunol.0903657 21115737 PMC3129036

[31] Fan QR Mosyak L Winter CC Wagtmann N Long EO Wiley DC. Structure of the inhibitory receptor for human natural killer cells resembles haematopoietic receptors. Nature (1997) 389(6646):96–100. 10.1038/38028 9288975

[32] Hilton HG Guethlein LA Goyos A Nemat-Gorgani N Bushnell DA Norman PJ Polymorphic HLA-C receptors balance the functional characteristics of KIR haplotypes. J Immunol (2015) 195(7):3160–70. 10.4049/jimmunol.1501358 26311903 PMC4575877

[33] Rajagopalan S Bryceson YT Kuppusamy SP Geraghty DE van der Meer A Joosten I Activation of NK cells by an endocytosed receptor for soluble HLA-G. PLoS Biol (2006) 4(1):e9. 10.1371/journal.pbio.0040009 16366734 PMC1318474

